# EV-Finder: Direct Detection of Extracellular Vesicle-Associated Proteins by Proximity Extension Assay for Multi-Cancer Screening

**DOI:** 10.3390/ijms27114904

**Published:** 2026-05-28

**Authors:** Yoshitaka Tamai, Fumiko Chiwaki, Yurika Shiotani, Hye-Eun Park, Eun-Jung Jung, Myung-Geun Shin, Young-Eun Lee, Yusuke Yoshioka, Takahiro Ochiya, Taek-Rim Yoon

**Affiliations:** 1 Medical Institute for Translational Health Science, ASFREYA Inc., 2-3-8 Shinkiba, Koto-ku, Tokyo 136-0082, Japan; ytamai@asfreya.co.jp (Y.T.); chiwaki@asfreya.co.jp (F.C.); yshiotani@asfreya.co.jp (Y.S.); 2 R&D Center, Exopia Co., Ltd., Room 210, Advanced Precision Medicine Industrialization Support Center, Chonnam National University Hwasun Hospital, 322, Seoyang-ro, Hwasun-eup, Hwasun-gun 58128, Republic of Korea; gracephe@naver.com (H.-E.P.); im1190@naver.com (E.-J.J.); 3 Department of Laboratory Medicine, Chonnam National University Medical School, Chonnam National University Hwasun Hospital, 322, Seoyang-ro, Hwasun-eup, Hwasun-gun 58128, Republic of Korea; mgshin@chonnam.ac.kr (M.-G.S.); dudmsgurwns@naver.com (Y.-E.L.); 4 Department of Molecular and Cellular Medicine, Institute of Medical Science, Tokyo Medical University, 6-1-1 Shinjuku, Shinjuku-ku, Tokyo 160-8402, Japan; yyoshiok@tokyo-med.ac.jp; 5 Department of Orthopedic Surgery, Chonnam National University Hwasun Hospital, 322, Seoyang-ro, Hwasun-eup, Hwasun-gun 58128, Republic of Korea

**Keywords:** extracellular vesicles, liquid biopsy, multi-cancer screening, EV-associated proteins, machine learning

## Abstract

Early cancer detection using minimally invasive biomarkers remains a significant challenge, particularly in early-stage disease, where circulating tumor DNA is often below the limit of detection. Extracellular vesicles (EVs), which are actively secreted by viable cancer cells and carry tumor-associated proteins, represent a promising alternative target for liquid biopsy. In this study, we developed EV-finder^®^, a conceptual framework for the direct detection of EV-associated proteins in serum using proximity extension assay (PEA) technology. Unlike conventional EV-based analytical methods that require prior EV isolation or enrichment, the EV-finder approach enables direct profiling of EV-associated proteins from small serum volumes without an EV isolation step, thereby simplifying the analytical workflow while preserving EV-derived molecular information. Using serum samples from patients with five cancer types (*n* = 193) and independent healthy controls (*n* = 138), we established a two-step supervised machine learning framework for cancer detection and tissue-of-origin prediction. The screening model demonstrated promising discriminative performance, with an AUC of 0.985, sensitivity of 0.929, and specificity of 0.957. Notably, no false positives were observed in an external Japanese control cohort, whereas 4 of 29 Korean control samples were classified as cancer-positive. Analysis of EV-associated protein profiles identified both pan-cancer and cancer-type-specific signatures, supporting their value for multi-cancer detection. Collectively, these findings demonstrate the potential feasibility of direct detection of EV-associated proteins from serum using PEA technology and highlight its potential as a scalable and minimally invasive strategy for multi-cancer screening.

## 1. Introduction

Early detection of cancer is a key determinant of improved clinical outcomes. However, currently available screening strategies are largely organ-specific, invasive, or limited by insufficient sensitivity for early-stage disease. Consequently, blood-based multi-cancer screening approaches have emerged as promising strategies for population-level cancer screening.

Circulating tumor DNA (ctDNA) has been extensively investigated as a liquid biopsy biomarker; however, its utility for early cancer detection remains limited. Because ctDNA is predominantly released as a consequence of tumor cell apoptosis or necrosis, its abundance is often low or undetectable in patients with early-stage disease, thereby limiting its sensitivity for early-stage cancer screening. These limitations highlight the need for alternative biomarkers that more faithfully reflect early tumor biology [1,2,3].

Extracellular vesicles (EVs) are membrane-bound nanoparticles actively secreted by viable cells, including cancer cells, throughout tumor development. EVs carry diverse biomolecular cargo, including proteins, nucleic acids, and lipids, that reflect the molecular characteristics of their cells of origin. Importantly, EVs are actively secreted by viable cancer cells, even during early-stage and pre-invasive lesions, highlighting the potential of EV-associated proteins as informative biomarkers for early cancer detection [4,5,6,7].

Recent advances in high-sensitivity proteomic technologies have enabled multiplexed quantification of circulating proteins from limited biological samples. Among these platforms, proximity extension assay (PEA) provides highly sensitive and specific protein detection through the use of paired antibodies conjugated to complementary DNA oligonucleotides, followed by real-time PCR amplification [8,9]. Despite these advantages, most EV-based analytical approaches still rely on EV isolation or enrichment prior to downstream molecular analysis. Such preprocessing procedures may introduce technical variability and limit the scalability and reproducibility required for clinical screening applications.

In the present study, we developed EV-finder^®^, a conceptual analytical framework that enables direct detection of EV-associated proteins from serum using PEA technology without prior EV isolation. Using this approach, we established a two-step machine learning model for cancer detection and cancer-type prediction based on EV-associated protein profiles.

## 2. Results

### 2.1. Concept and Principle of the EV-Finder Assay

EVs released by cancer cells circulate in the bloodstream and can be detected in serum obtained through standard blood collection and centrifugation. However, the highly sensitive detection of EV-associated proteins from very small sample volumes remains technically challenging.

**Figure 1:**
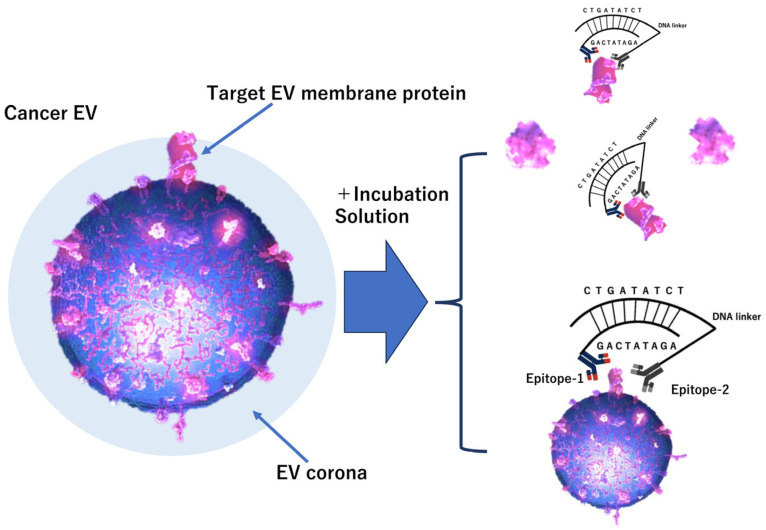
Schematic illustration of the EV-finder assay. Serum extracellular vesicles (EVs) are detected using antibody–DNA conjugates based on the proximity extension assay (PEA) principle. When two antibodies bind in close proximity to the same target protein, the attached DNA oligonucleotides hybridize, enabling subsequent amplification and quantification by real-time PCR.

In this study, we developed the EV-finder framework, a conceptual strategy that integrates extracellular vesicle biology with PEA technology to enable ultrasensitive detection of EV-associated proteins directly from serum (Figure 1).

The Olink PEA platform was used for high-throughput protein profiling. In the EV-finder assay, 1 μL of serum containing circulating EVs was incubated with the proprietary incubation solution provided by Olink together with antibody–DNA conjugates. When two antibodies simultaneously bind to the same target protein, the attached DNA oligonucleotides are brought into proximity, enabling hybridization and generation of a DNA template for real-time PCR-based amplification and quantification.

Although the detailed composition of the incubation solution has not been publicly disclosed by Olink, the assay conditions permit antigen–antibody interactions with EV-associated proteins. Two possible mechanisms may account for this process: (i) partial solubilization of EV membranes, resulting in the exposure of membrane-associated proteins, or (ii) direct binding of antibodies to membrane proteins present on the surface of intact EVs.

We observed no clear evidence of gross EV lysis induced by the incubation solution (Supplementary Materials), indicating that EV-associated proteins can be detected under these assay conditions.

By conceptually integrating the ultrasensitive PEA platform with EV-associated protein detection, the EV-finder assay enables high-throughput, sensitive measurement of EV-associated proteins from very small volumes of biological samples.

### 2.2. EV-Associated Protein Expression Patterns Across Cancer Types

**Figure 2:**
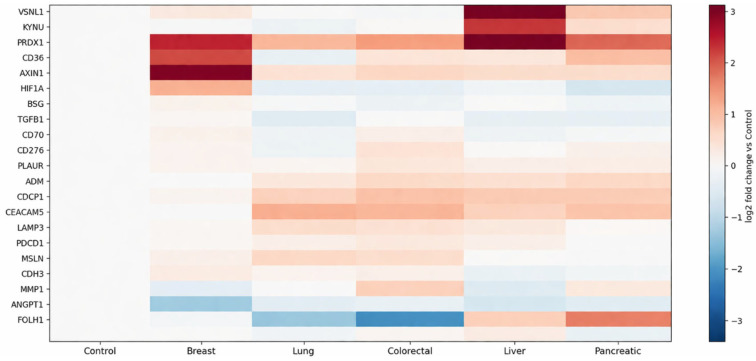
Heatmap of 21 EV-associated proteins across five cancer types relative to control samples. Protein expression levels measured using the Olink proximity extension assay (PEA) are presented as log_2_ fold change relative to the median expression level of the control group. Red indicates increased expression, blue indicates decreased expression, and white indicates minimal or no change. Proteins are ordered by hierarchical clustering based on similarities in expression patterns across cancer types. The control group is included as a reference column, with the log_2_ fold change set to 0.

To investigate global differences in EV-associated protein expression across cancer types, the relative expression levels of 21 EV-associated proteins were visualized using a heatmap (Figure 2).

Protein expression values are presented as log_2_-transformed fold changes relative to the median expression level of the control group.

Heatmap analysis revealed both shared and cancer-type-specific expression patterns across the five cancer types examined. Several proteins showed increased expression in multiple cancer types. In particular, PRDX1, CD36, and AXIN1 were upregulated in several tumor types relative to controls, suggesting the presence of EV-associated protein alterations common across cancers.

In contrast, certain proteins exhibited cancer-type-specific expression patterns. VSNL1 and KYNU showed prominent upregulation in liver cancer samples, whereas CEACAM5 displayed increased expression in lung cancer. These patterns suggest that EV protein expression profiles may reflect tissue-specific tumor biology.

Conversely, several proteins showed reduced expression relative to the control group. Notably, ANGPT1 and FOLH1 were downregulated in multiple cancer types, with particularly marked reductions in lung and colorectal cancers.

To facilitate interpretation of these findings, a categorical summary of the relative expression behavior of the ten EV-associated proteins across the five cancer types is provided in Supplementary Table S3. Protein expression changes were classified according to fold-change thresholds based on the ratio of median protein levels in each cancer group relative to the control group. Whereas Figure 2 presents these differences as log_2_ fold changes, Supplementary Table S3 provides a simplified categorical representation to facilitate intuitive comparison of protein expression patterns among cancer types.

Overall, these findings suggest that EV-associated protein expression profiles encode both pan-cancer and cancer-type-specific information, underscoring their potential for multi-cancer detection and cancer-type classification.

### 2.3. Overview of the Two-Step EV-Finder Algorithm

**Figure 3:**
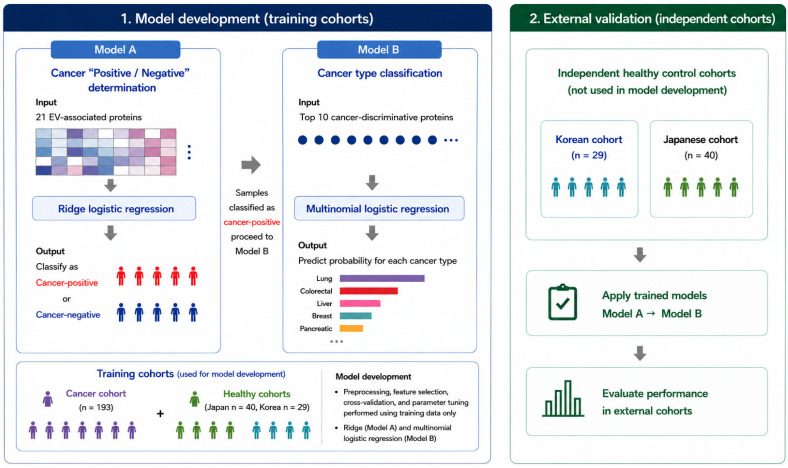
Overview of the EV-finder two-step machine learning framework. Serum EV-associated protein profiles were measured using the proximity extension assay (PEA). In Step 1, Model A applied ridge logistic regression to classify samples as cancer-positive or cancer-negative based on 21 EV-associated proteins. In Step 2, Model B applied multinomial logistic regression to samples classified as cancer-positive in Step 1 using the top 10 cancer-discriminative proteins, thereby estimating the most likely cancer type. Independent healthy control cohorts were reserved for external validation and were entirely excluded from feature selection, parameter optimization, cutoff determination, and model training.

To achieve sensitive cancer detection and accurate cancer-type classification, we established a two-step analytical framework termed EV-finder (Figure 3).

In the first step (Model A), ridge logistic regression was employed to classify serum samples as cancer-positive or cancer-negative based on EV-associated protein expression profiles. Ridge regularization was applied to stabilize model coefficients and reduce the effects of multicollinearity among EV-associated proteins.

In the second step (Model B), samples predicted as cancer-positive by Model A were further subjected to cancer-type classification using multinomial ridge logistic regression. This model estimated the probability of each of the five cancer types—breast, lung, colorectal, liver, and pancreatic cancers—based on EV-associated protein expression patterns.

For Model B, ten EV-associated proteins were selected from the training dataset using one-way analysis of variance (ANOVA) across the five cancer types, and candidate proteins were ranked according to the F-statistic.

By separating cancer detection from cancer-type classification, this two-step framework allows independent optimization of cancer screening and cancer-type classification while reducing the influence of low-confidence samples on downstream cancer-type prediction.

### 2.4. Model A Performance for Cancer Screening

The performance of Model A for cancer screening was assessed using ridge logistic regression applied to the expression profiles of 21 EV-associated proteins. The model was trained on cancer samples from the Korean cohort and healthy control samples from the designated training cohorts.

**Figure 4:**
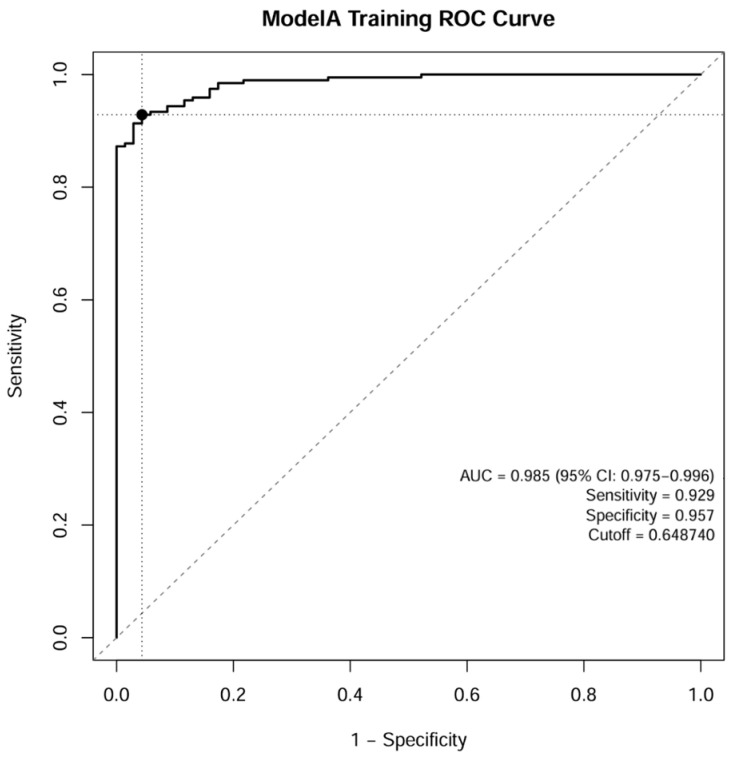
Performance of the cancer screening model, Model A. Receiver operating characteristic (ROC) curve for Model A. The model achieved an area under the ROC curve (AUC) of 0.9854. The optimal cutoff value of 0.6487 was determined using the Youden index, yielding a sensitivity of 0.929 and a specificity of 0.957.

In the training dataset, Model A showed excellent discrimination between cancer and non-cancer samples, with an area under the receiver operating characteristic curve (AUC) of 0.985 (95% CI, 0.975–0.996) (Figure 4).

The optimal cutoff value for cancer positivity was determined using the Youden index, resulting in a sensitivity of 0.929 and a specificity of 0.957 in the training dataset. The probability threshold was 0.6487.

To evaluate the generalizability of the screening model, external validation was conducted using independent healthy control cohorts from Japan and Korea. Notably, the same fixed cutoff derived from the training dataset was applied to both external cohorts without modification. Because these external cohorts consisted exclusively of healthy individuals, the analysis focused on assessing the false-positive rate of the screening model in independent populations.

In the external validation analysis, no false-positive results were observed in the Japanese healthy control cohort (*n* = 40), corresponding to a specificity of 1.000. In contrast, four samples in the Korean healthy cohort (*n* = 29) were classified as cancer-positive (Table 1).

Overall, these results indicate that the EV-associated protein-based screening model provides accurate discrimination between cancer and non-cancer samples while maintaining high specificity across independent validation cohorts.

### 2.5. Cancer-Type Prediction Using Model B

Samples identified as cancer-positive by Model A were subsequently analyzed using Model B to estimate the probability of each cancer type. Model B employed multinomial ridge logistic regression based on EV-associated protein expression profiles and was trained exclusively on cancer samples.

Among the external healthy control samples from the Korean cohort, four were classified as cancer-positive by Model A. These samples were subsequently subjected to cancer-type prediction using Model B.

As shown in Table 2, the predicted cancer types varied among these samples. Lung cancer was the top-ranked prediction in two samples, whereas colorectal cancer was the top-ranked prediction in the remaining two.

The probability scores ranged from 0.362 to 0.484, reflecting the relative likelihoods assigned by the multinomial classification model. In each sample, the second-ranked cancer type also showed a substantial probability, indicating that these outputs should be interpreted as probabilistic predictions across multiple cancer types rather than definitive diagnostic assignments.

Importantly, these findings demonstrate that the EV-finder framework can generate probabilistic cancer-type predictions for samples identified as cancer-positive by the screening model, underscoring the potential of EV-associated protein signatures for multi-cancer classification.

## 3. Discussion

This study demonstrates the potential feasibility of the EV-finder framework, a two-step EV-based machine learning strategy for multi-cancer screening using serum EV-associated protein profiles [4]. By integrating ultrasensitive PEA technology with EV-associated protein profiling, EV-finder enables the detection of cancer-associated EV signals from very small serum volumes. The cancer screening model (Model A) achieved promising discriminative performance, with an area under the ROC curve (AUC) of 0.985 in the training dataset. Notably, when the same fixed decision threshold derived from the training dataset was applied to independent healthy cohorts, no false-positive results were observed in the Japanese healthy control cohort. Although these findings support the potential robustness of the framework under carefully controlled pre-analytical conditions, further validation in larger independent cohorts will be necessary to assess the reproducibility and generalizability of the approach.

In liquid biopsy research, it is widely recognized that, beyond the analytical platform itself, rigorous control of pre-analytical conditions following sample collection is essential for reproducibility and appropriate interpretation of results [10,11,12,13,14]. For blood-based biomarkers, factors such as blood collection procedures, sample processing protocols, hemolysis, storage temperature, and storage duration may substantially influence measured molecular profiles [13,14,15]. Variability in pre-analytical conditions may alter baseline molecular signatures in healthy control populations and potentially contribute to false-positive findings or overestimation of biomarker performance.

Similar challenges have also been reported in EV-based liquid biopsy studies. Differences in blood processing conditions and EV isolation or recovery methods may directly influence analytical outcomes [10]. In addition, the presence of a protein corona surrounding EVs may interfere with antigen–antibody interactions, thereby affecting the detection of EV surface markers [16]. Because EVs represent a heterogeneous population with diverse cellular origins and physicochemical properties, the choice of separation and enrichment methods, as well as technical handling conditions, may substantially affect the EV subpopulations detected and their associated molecular cargo [17]. Accordingly, the EV research community has consistently emphasized the importance of clearly documenting sample preparation and EV recovery methods and interpreting findings with careful consideration of methodological differences across studies [18].

Consistent with these considerations, the present study applied harmonized pre-analytical protocols across participating institutions, including standardized procedures for blood collection, serum separation, and storage. Nevertheless, measurable differences in assay results were observed between the Japanese and Korean healthy control cohorts. A relatively higher proportion of samples in the Korean cohort were classified as cancer-positive by Model A. At present, these cases are conservatively interpreted as false positives because all individuals were clinically classified as healthy at the time of sample collection. However, the possibility that EV-associated protein signatures may capture broader biological variation not reflected in routine clinical assessments cannot be excluded.

Factors including age distribution, lifestyle-related exposures (such as smoking and dietary habits), metabolic or inflammatory status, ethnic background, and other physiological variables may influence circulating EV-associated protein signatures and contribute to elevated cancer-risk scores in individuals without clinically diagnosed malignancy. In addition, detailed clinical metadata, including smoking status, inflammatory conditions, and longitudinal follow-up information, were not uniformly available across all cohorts in the present study. Accordingly, variability among healthy control groups likely reflects a combination of pre-analytical factors and subject-related biological differences rather than a single dominant cause. Further clarification of this issue will require larger multicenter studies incorporating comprehensive clinical metadata, age- and sex-matched control populations, lifestyle information, and prospective longitudinal follow-up for cancer incidence.

In addition to supporting the proof-of-concept feasibility of EV-based cancer screening, the present study also provides preliminary insight into the biological characteristics of EV-associated protein signatures. Heatmap analysis revealed both shared pan-cancer alterations and cancer-type-related expression patterns. Several markers demonstrated consistent expression changes across multiple cancer types, suggesting that common tumor-associated biological processes may contribute to EV-associated protein alterations regardless of tissue origin. These markers may partly contribute to the promising performance of the pan-cancer detection model (Model A).

Importantly, ANGPT1 consistently showed reduced expression in cancer-derived EVs relative to controls across multiple cancer types. This observation suggests that pan-cancer EV signatures are not restricted to proteins upregulated in cancer but may also include proteins downregulated in association with malignancy. This finding highlights the importance of considering both directions of expression change when developing EV-based screening models.

Conversely, other proteins showed expression changes that appeared to be more specific to individual cancer types. These cancer-type-dependent proteins likely reflect tissue-specific tumor biology and may provide additional discriminatory information for cancer-type classification. In the EV-finder framework, such proteins are incorporated into the second analytical step (Model B), which estimates the relative probabilities of different cancer types using multinomial logistic regression.

Cancer-type prediction using Model B showed promising but still preliminary trends in the present dataset. This limitation likely reflects both the relatively small sample size and the biological heterogeneity among different cancer types, which remains a common challenge in multi-cancer detection studies [17,19,20]. The multinomial classification model provides probabilistic estimates for each cancer type rather than definitive diagnostic assignments, and several samples showed relatively similar probability values across multiple cancer types. This probabilistic behavior likely reflects both the limited sample size available for training and the intrinsic biological overlap among EV-associated protein signatures derived from different tumor types. Similar challenges in distinguishing cancer types from blood-based molecular signals have been reported in multi-cancer early detection studies [20]. In addition, certain cancers, including pancreatic cancer, are known to exhibit substantial biological heterogeneity and diagnostic complexity even in conventional clinical settings [21]. Accordingly, cancer-type prediction within the present framework should be regarded as exploratory and proof-of-concept rather than as a definitive diagnostic output.

Compared with existing multi-cancer screening approaches, including ctDNA-based methods, the EV-finder framework may offer several potential advantages. Cancer cells actively release EVs into circulation, potentially enabling detection of tumor-derived molecular signals even at early stages of disease [2,4,6]. In addition, the PEA-based analytical platform allows highly sensitive measurement of multiple protein targets from very small sample volumes, providing a scalable strategy for multiplexed EV biomarker detection.

Despite these promising findings, several important limitations must be acknowledged. First, the present study included a relatively limited number of samples, and the cancer types analyzed were restricted to five major malignancies. Second, although no clear evidence of gross EV lysis was observed under the present experimental conditions, the precise molecular origin of the detected signals requires further investigation. The relative contribution of vesicle-associated proteins, soluble serum proteins, and partially disrupted vesicles could not be fully distinguished in the current study. Future mechanistic studies will therefore be necessary to further clarify the biological basis of the detected signals. Third, the current study design was retrospective and exploratory in nature. Future studies involving larger multicenter cohorts, additional cancer types, and prospective longitudinal follow-up will be required to evaluate the reproducibility, generalizability, and potential clinical utility of the EV-finder framework.

Taken together, these findings support the proof-of-concept feasibility of using EV-associated protein signatures measured by ultrasensitive PEA technology for cancer detection and exploratory cancer-type prediction within a unified analytical framework. With additional validation, methodological standardization, and prospective multicenter evaluation, the EV-finder strategy may represent a promising minimally invasive approach for future multi-cancer screening applications based on circulating EVs.

## 4. Materials and Methods

### 4.1. Serum Sample Collection

Blood samples were collected from all participants in accordance with protocols approved by the Institutional Review Board (IRB) of the participating institutions (approval number: ASF001-2025 and CNUHH-2023-047). The study was conducted in compliance with the principles of the Declaration of Helsinki. Written informed consent was obtained from all participants prior to blood collection.

Peripheral blood samples (5 mL) were collected from each participant by standard venipuncture. Serum samples were prepared using standard serum separation procedures, including clotting at room temperature and subsequent centrifugation to remove cellular components. The resulting serum samples were aliquoted and stored at −80 °C until further analysis.

### 4.2. Study Cohorts and Serum Samples

The study was approved by the Institutional Review Board of ASFREYA Inc. for secondary analysis (ASF001-2026). Blood collection was approved by the Institutional Review Boards of ASFREYA Inc. (ASF001-2025, approved on 3 September 2025) and Chonnam National University Hwasun Hospital (CNUHH-2023-047, approved on 21 February 2023). All participants provided written informed consent. Serum samples were obtained from two independent sources. The cancer cohort consisted of patients with five cancer types—lung, colorectal, liver, breast, and pancreatic cancers—whose serum samples were collected at the Biobank of Chonnam National University Hwasun Hospital, a member of the Korea Biobank Network. Each cancer type included 40 patients, except for pancreatic cancer (*n* = 33), resulting in a total of 193 cancer samples.

Healthy control samples were obtained from two independent cohorts. In total, 138 serum samples were collected from healthy individuals, including 80 samples from the IIT Clinic in Tokyo, Japan, and 58 samples from Chonnam National University in Korea.

The 80 healthy serum samples collected in Japan were divided into two balanced sub-cohorts of 40 samples each to generate independent training and validation datasets. In addition, 58 healthy serum samples were obtained from a Korean cohort collected at the same institution as the cancer samples. These samples were similarly divided into two sub-cohorts of 29 samples each.

For model development, one Japanese sub-cohort (*n* = 40) and one Korean sub-cohort (*n* = 29) were used as healthy control samples together with the cancer cohort for model training. The remaining Japanese sub-cohort (*n* = 40) and the remaining Korean sub-cohort (*n* = 29) were reserved exclusively for external validation and were not used during model development. All data preprocessing steps, including normalization and missing-value imputation, were performed using parameters estimated from the training dataset and subsequently applied to the validation datasets to avoid information leakage.

Independent external validation cohorts were completely separated from all stages of model development, including feature selection, parameter optimization, threshold determination, and model fitting. Cross-validation procedures were conducted exclusively within the training datasets and did not involve samples from the external validation cohorts.

### 4.3. Measurement of EV-Associated Proteins

EV-associated protein levels were measured using the Olink^®^ Flex platform based on proximity extension assay (PEA) technology (Olink Proteomics AB, Uppsala, Sweden) (Figure 1), according to the manufacturer’s instructions.

A customized panel consisting of 21 proteins was designed using the Olink^®^ Flex system. The target proteins were selected from the Olink protein panel library based on prior exploratory analyses and their biological relevance to extracellular vesicle-associated cancer signaling.

Protein expression levels were reported as normalized protein expression (NPX) values on a log_2_ scale. Internal normalization, inter-plate normalization, and quality control were performed according to the manufacturer’s standard protocols. Samples that did not meet quality control criteria were excluded from further analysis. All measurements were carried out using standardized assay procedures in accordance with the manufacturer’s recommended guidelines.

To minimize potential batch effects, harmonized sample collection, serum processing, storage, and analytical workflows were applied across participating institutions whenever possible. In addition, internal normalization and inter-plate normalization incorporated within the Olink^®^ platform were used to reduce technical variability across assay runs.

For each cancer type, expression levels of the 21 EV-associated proteins were compared between cancer and control samples using the Mann–Whitney U test. To account for multiple testing, *p*-values were adjusted using the Benjamini–Hochberg false discovery rate (FDR) correction.

Global expression patterns among cancer types were visualized using a heatmap based on relative protein expression levels (Figure 2).

### 4.4. Overview of the Two-Step Algorithm

The EV-finder framework consists of two sequential analytical steps designed to separate cancer screening from cancer-type identification. In Step 1 (Model A), cancer screening was performed using ridge logistic regression to classify serum samples as cancer-positive or cancer-negative based on EV-associated protein expression levels.

Ridge regularization was applied to stabilize model coefficients and mitigate multicollinearity among EV-associated proteins. The regularization parameter was determined using cross-validation. Model training was performed using cancer samples together with a subset of healthy control samples from the training cohorts, and the optimal decision threshold was determined using the Youden index.

To reduce the risk of overfitting and improve model robustness, ridge regularization was incorporated into both Model A and Model B. Cross-validation procedures were conducted exclusively within the training datasets, and external validation cohorts were not used during parameter optimization, threshold determination, or feature selection.

In Step 2 (Model B), cancer-type identification was performed exclusively for samples classified as cancer-positive in Step 1. Multinomial logistic regression was applied to estimate the probability of each cancer type based on EV-associated protein expression levels, and predicted cancer types were ranked according to their predicted probabilities.

This two-step design allows independent optimization of cancer screening and cancer-type classification while reducing the impact of uncertain screening results on downstream cancer-type prediction. A schematic overview of the workflow is provided in Figure 3.

### 4.5. EV-Associated Protein Selection

For Model A, all 21 EV-associated proteins were used as input features. These proteins were selected based on prior evidence demonstrating their presence in extracellular vesicles derived from cancer tissues, as reported in publicly available EV databases and previous studies.

### 4.6. Model A: Cancer Screening

Ridge logistic regression was used to classify serum samples as cancer-positive or cancer-negative while mitigating multicollinearity among EV-associated proteins and improving model stability. The model was trained using cancer samples from the Korean cohort together with the integrated healthy control training cohort.

Prior to model training, missing values in EV-associated protein measurements were imputed using the median value of each protein calculated from the training dataset. The data were subsequently standardized by centering and scaling using the parameters estimated from the training dataset, and the same preprocessing parameters were applied to the external validation datasets.

Model training was performed using ridge logistic regression implemented in the glmnet package in R (alpha = 0). The optimal regularization parameter (λ) was determined using cross-validation, and the model corresponding to the minimum cross-validation error (λ_min) was selected. Cross-validation procedures were restricted to the training datasets in order to prevent information leakage into the independent validation cohorts.

The optimal cutoff value for cancer positivity was determined using the Youden index, which maximizes the sum of sensitivity and specificity based on the training receiver operating characteristic (ROC) curve.

Cancer status used as the ground-truth label was determined based on clinical diagnosis and pathological confirmation documented in the corresponding medical records.

### 4.7. Model B: Cancer-Type Classification

For samples classified as cancer-positive by Model A, cancer-type classification was performed using multinomial ridge logistic regression. The model was designed to discriminate among five cancer types (lung, colorectal, liver, breast, and pancreatic cancers) based on EV-associated protein expression profiles.

Feature selection for Model B was carried out using only the cancer samples in the training dataset. For each EV-associated protein, one-way analysis of variance (ANOVA) was performed across the five cancer types, and proteins were ranked according to the F-statistic. The 10 proteins with the highest F-values were selected as input features for Model B.

Missing data points were imputed using protein-wise median values from the training dataset, followed by standardization using parameters estimated from the training dataset.

Multinomial ridge logistic regression was implemented using the glmnet package in R (alpha = 0). The optimal regularization parameter (λ) was determined by cross-validation, and the model corresponding to the minimum mean cross-validation error (λ_min, as defined in the glmnet package) was selected.

For each sample, the model estimated the probability of belonging to each of the five cancer types. Predicted cancer types were ranked based on these probability scores, and the top-ranked predictions were used for interpretation of cancer-type classification results.

Only samples classified as cancer-positive by Model A were subjected to Model B analysis in order to reduce the influence of low-confidence samples on cancer-type prediction.

### 4.8. Statistical Analysis

Statistical analyses were performed to evaluate the performance of the cancer screening and cancer-type classification models. Receiver operating characteristic (ROC) curves were generated, and the area under the ROC curve (AUC) was calculated to assess overall discriminative performance. Sensitivity, specificity, and false-positive rates were calculated based on the predefined cutoff values for both the training dataset and independent external validation cohorts.

All statistical analyses and machine learning procedures were conducted using R statistical software (version 4.4.3; R Foundation for Statistical Computing, Vienna, Austria) and RStudio IDE (version 2025.05.1+513; Posit Software, PBC, Boston, MA, USA).

## 5. Conclusions

In conclusion, this study supports the proof-of-concept feasibility of the EV-finder framework, a two-step machine learning strategy for multi-cancer screening based on EV-associated protein profiles measured using ultrasensitive PEA technology. By enabling the detection of EV-associated protein signals from very small serum volumes, this framework may provide a scalable platform for high-throughput liquid biopsy analysis. The cancer screening model (Model A) demonstrated promising discrimination performance and showed relatively stable behavior when applied to independent healthy cohorts using a fixed decision threshold. In addition, exploratory cancer-type prediction using multinomial classification (Model B) suggested that EV-associated protein signatures may contain both shared pan-cancer signals and cancer-type-related patterns.

However, cancer-type prediction remains preliminary and requires additional validation in substantially larger and more diverse datasets. The hierarchical modeling strategy may nevertheless offer an advantage by allowing independent optimization of cancer detection and cancer-type estimation. Future studies involving larger multicenter cohorts, expanded cancer types, standardized pre-analytical protocols, and prospective longitudinal follow-up will be essential to evaluate the reproducibility, generalizability, and potential clinical applicability of this framework.

Overall, with additional validation and methodological refinement, EV-finder may represent a promising minimally invasive approach for future population-level multi-cancer screening based on circulating EV-associated protein signatures.

## Figures and Tables

**Table 1:** External validation results of Model A in independent healthy cohorts. Model A was trained using cancer samples from a Korean cohort together with healthy control samples from Japanese and Korean cohorts. A fixed cutoff value of 0.6487, determined in the training cohort using the Youden index, was applied to two independent external healthy cohorts. Specificity was calculated for each cohort based on the number of samples classified as false positives.

Cohort	*n*	Cutoff	False Positives (*n*)	Specificity (%)	Notes
Japanese	40	0.6487	0	100	No false positives observed
Korean	29	0.6487	4	86.2	Subset showed high cancer-like scores

**Table 2:** Cancer-type prediction results among samples classified as cancer-positive. Samples from the Korean healthy control cohort classified as cancer-positive by Model A were subsequently analyzed using Model B to estimate cancer-type probabilities. For each sample, predicted probabilities for the five cancer types are presented together with the two highest-ranked predicted cancer types, denoted as B_top1 and B_top2. Because Model B was trained exclusively on cancer samples, these probabilities should be interpreted as relative likelihoods across the evaluated cancer types rather than as absolute diagnostic probabilities.

Sample ID	A_Probability	B_Top1	B_Top1_Probability	B_Top2	B_Top2_Probability
#12	0.6973	Colorectal	0.3624	Pancreatic	0.2831
#20	0.8812	Lung	0.4709	Pancreatic	0.2385
#22	0.8866	Lung	0.384	Colorectal	0.2632
#N3	0.6548	Colorectal	0.4835	Lung	0.2494

## Data Availability

The datasets generated and/or analyzed during the current study are not publicly available due to proprietary and commercial considerations but may be made available from the corresponding author upon reasonable request, subject to appropriate agreements.

## Supplementary Materials

The following supporting information can be downloaded at: https://www.mdpi.com/article/10.3390/ijms27114904/s1.
