# Supplementary material for: EV-Finder: Direct Detection of Extracellular Vesicle-Associated Proteins by Proximity Extension Assay for Multi-Cancer Screening
Source: Int J Mol Sci. 2026 May 28;27(11):4904. doi: 10.3390/ijms27114904 (PMC13256567; doi:10.3390/ijms27114904)
Supplement: Supplementary file 1 [file ijms-27-04904-s001.zip › ijms-4302072-supplementary.pdf]

**Supplementary Material**

**Table S1.** Particle concentration of serum samples measured by Nanoparticle Tracking Analysis (NTA)

| | Pre-incubation particle count | Post-incubation particle count | ΔN (particle count difference) | Relative change |
|---|---|---|---|---|
| Olink Training Sample Serum | 4.74 × 10^11 | 5.17 × 10^11 | +4.32 × 10^10 | +9.07% |
| Pancreatic cancer serum | 3.11 × 10^11 | 1.26 × 10^11 | −1.85 × 10^11 | −59.49% |
| Lung cancer serum | 1.90 × 10^11 | 1.72 × 10^11 | −1.78 × 10^10 | −9.47% |
| Liver cancer serum | 4.54 × 10^11 | 2.34 × 10^11 | −2.20 × 10^11 | −48.46% |
| Colorectal cancer serum | 7.38 × 10^11 | 3.58 × 10^11 | −3.80 × 10^11 | −51.49% |
| Breast cancer serum | 3.99 × 10^11 | 4.21 × 10^11 | +2.22 × 10^10 | +5.51% |

An increase was observed in the breast cancer sample following Olink incubation, whereas a slight decrease (~10%) was observed in the lung cancer sample. Marked decreases (~48–60%) were observed in the pancreatic, liver, and colorectal cancer samples. Because the responses varied among samples, these results do not support the conclusion that the incubation solution causes uniform particle degradation.

**Table S2.** Particle size distribution of serum samples measured by Nanoparticle Tracking Analysis (NTA)

| | Pre-incubation particle size | Post-incubation particle size | Δsize (particle size difference) | Relative change |
|---|---|---|---|---|
| Olink Training Sample Serum | 80.9 nm | 82.2 nm | +1.3 nm | +1.61% |
| Pancreatic cancer serum | 62.4 nm | 67.4 nm | +5.0 nm | +8.01% |
| Lung cancer serum | 62.9 nm | 82.2 nm | +19.3 nm | +30.68% |
| Liver cancer serum | 73.3 nm | 64.2 nm | −9.1 nm | −12.41% |
| Colorectal cancer serum | 63.1 nm | 62.95 nm | −0.15 nm | −0.24% |
| Breast cancer serum | 64.5 nm | 65.0 nm | +0.5 nm | +0.78% |

No systematic reduction in particle size was observed across the samples. Particle size increased in the lung cancer sample (+30.68%) but decreased in the liver cancer sample (−12.41%). These results do not support a consistent reduction in particle size following incubation, indicating that the effects of the incubation solution are sample-dependent.

## Supplementary Methods

### *Detailed NTA measurement procedure*

The effect of the incubation mixture used in the Olink assay on serum particles was evaluated. Following the standard Olink assay conditions, an incubation mixture was prepared by combining Olink® 1–48 plex Incubation Solution, Olink® Flex Frw-probes, and Olink® Flex Rev-probes at a ratio of 8:1:1. Serum (1 µL) was added to 3 µL of the incubation mixture, vortexed to mix, and incubated overnight at 4 °C. As a solvent control, 1 µL of PBS(-) was added instead of serum.

Particle concentration and size distribution were measured using Nanoparticle Tracking Analysis (NTA) with a NanoSight NS300 system (Malvern Panalytical, Malvern, UK). Serum samples prior to incubation were diluted 1:1000 in PBS(-) and subjected to NTA measurement. After incubation, samples were diluted 250-fold in PBS(-) to standardize the final particle concentration. All samples were filtered through a 0.22 µm filter immediately prior to measurement.

NTA measurements were performed using a Blue 405 laser with the following settings: camera level 13, detection threshold 7, and syringe pump speed 40. Each sample was measured three times, and the average value was used as the representative measurement. Pre-incubation samples were measured once, whereas post-incubation samples were measured twice and the mean values were calculated.

**Table S3.** Relative expression patterns of the top 10 EV-associated proteins across five cancer types

| | Breast | Colorectal | Liver | Lung | Pancreatic |
|---|---|---|---|---|---|
| AXIN1 | ↑↑↑ | ↑↑ | ↑↑ | ↑ | ↑ |
| KYNU | → | → | ↑↑↑ | → | ↑ |
| CD36 | ↑↑↑ | ↑ | ↑ | ↓ | ↑↑ |
| VSNL1 | ↑ | → | ↑↑↑ | → | ↑↑ |
| PRDX1 | ↑↑↑ | ↑↑↑ | ↑↑↑ | ↑↑↑ | ↑↑↑ |
| ANGPT1 | ↓↓↓ | ↓ | ↓ | ↓ | ↓ |
| MMP1 | ↓ | ↑↑ | ↓ | → | ↑ |
| ADM | → | ↑ | ↑ | ↑ | ↑↑ |
| CD276 | → | ↑ | → | → | → |
| TGFB1 | → | → | ↓ | ↓ | ↓ |

Protein expression changes were evaluated relative to the control group, which consisted of combined Japanese and Korean samples. Fold changes were calculated as the ratio of the median protein level in each cancer group to that in the control group. Protein expression patterns were categorized according to fold change thresholds as follows: ↑↑↑ (≥ 2.0), ↑↑ (≥ 1.5), ↑ (≥ 1.2), → (0.83–1.2), ↓ (≤ 0.83), ↓↓ (≤ 0.67), ↓↓↓ (≤ 0.5). Arrows indicate the direction and magnitude of expression changes relative to the control group.

**Table S4.** Clinicopathological characteristics of patients for each cancer type

**(A) Liver cancer (Hepatocellular carcinoma)**

| Characteristic | Value |
|---|---|
| Number of patients (n) | 40 |
| Age, years (median [range]) | 62 [42–81] |
| Sex, n (Male / Female) | 35 / 5 |
| Histological diagnosis | Hepatocellular carcinoma |
| Early stage (T1–T2) | 27 |
| Advanced T3–T4 or lymph node–positive (N1) | 13 |

**(B) Colorectal cancer**

| Characteristic | Value |
|---|---|
| Number of patients (n) | 40 |
| Age, years (median [range]) | 73 [44–92] |
| Sex, n (Male / Female) | 22 / 18 |
| Early stage (T1–T2) | 0 |
| Advanced T3–T4 or lymph node–positive (N1) | 40 |

**(C) Breast cancer**

| Characteristic | Value |
|---|---|
| Number of patients (n) | 40 |
| Age, years (median [range]) | 58 [29–78] |
| Sex, n (Female / Male) | 40 / 0 |
| Early stage (T1–T2) | 29 |
| Advanced T3–T4 or lymph node–positive (N1) | 11 |

**(D) Lung cancer**

| Characteristic | Value |
| --- | --- |
| Number of patients (n) | 40 |
| Age, years (median [range]) | 72 [50–84] |
| Sex, n (Male / Female) | 26 / 14 |
| Early stage (T1–T2) | 28 |
| Advanced T3–T4 or lymph node–positive (N1) | 12 |

**(E) Pancreatic cancer**

| Characteristic | Value |
| --- | --- |
| Number of patients (n) | 33 |
| Age, years (median [range]) | 69 [36–86] |
| Sex, n (Male / Female) | 19 / 14 |
| Early stage (T1–T2) | 19 |
| Advanced T3–T4 or lymph node–positive (N1) | 14 |

Clinicopathological characteristics of the cancer patients included in this study are summarized in Table S4. Age is presented as median with range. Patients in the cancer cohort were stratified into early and advanced groups using a simplified TNM-based definition. Early-stage disease was defined as T1–T2, whereas advanced-stage disease was defined as T3–T4 or lymph node–positive (N1) tumors. Stage-stratified analysis was not performed for colorectal cancer due to the lack of early-stage cases. This distribution reflects the clinical reality of colorectal cancer diagnosis, where early-stage tumors are often underrepresented in surgically resected cohorts.
One limitation of this study is the lack of demographic information, including age and sex, for the healthy control cohort. However, the primary objective was to evaluate biomarker performance in distinguishing cancer from non-cancer samples. The observed differences are unlikely to be solely explained by demographic factors, given their consistency across multiple cancer types.
